# Asynchronous Video Interviewing as a New Technology in Personnel Selection: The Applicant’s Point of View

**DOI:** 10.3389/fpsyg.2016.00863

**Published:** 2016-06-14

**Authors:** Falko S. Brenner, Tuulia M. Ortner, Doris Fay

**Affiliations:** ^1^Institute of Psychology, Freie Universität BerlinBerlin, Germany; ^2^Department of Psychology, University of SalzburgSalzburg, Austria; ^3^Department of Psychology, University of PotsdamPotsdam, Germany

**Keywords:** applicant reactions, new technology, selection, asynchronous video interviewing, technology acceptance model

## Abstract

The present study aimed to integrate findings from technology acceptance research with research on applicant reactions to new technology for the emerging selection procedure of asynchronous video interviewing. One hundred six volunteers experienced asynchronous video interviewing and filled out several questionnaires including one on the applicants’ personalities. In line with previous technology acceptance research, the data revealed that perceived usefulness and perceived ease of use predicted attitudes toward asynchronous video interviewing. Furthermore, openness revealed to moderate the relation between perceived usefulness and attitudes toward this particular selection technology. No significant effects emerged for computer self-efficacy, job interview self-efficacy, extraversion, neuroticism, and conscientiousness. Theoretical and practical implications are discussed.

## Introduction

When it comes to new technology used for personnel selection, applicant reactions have piqued the interest of personnel researchers and practitioners (Anderson, 2003; Chapman et al., 2003; Bauer et al., 2004, 2006; Stoughton et al., 2015). The primary goals of implementing new technology for recruitment and selection are efficiency, cost reduction, system standardization, and extension of the applicant pool (Chapman and Webster, 2003). However, when pursuing these goals, potentially undesired effects on the applicants should also be taken into consideration. Applicant reactions are further associated with organizational attractiveness, intentions to accept a job offer, and the likelihood of recommending that others apply to this employer (Hausknecht et al., 2004; Truxillo et al., 2009). Therefore, organizations should not only aim to ensure that selection procedures will identify the best candidates, they also have to avoid adverse effects on their applicants.

The employment interview is still one of the most widely used selection methods. Interviews can also include delayed or asynchronous interaction (Levashina et al., 2014). Recorded or asynchronous video interviewing represents a newly emerging technology that can be used in the screening stage of a selection process. Job applicants are invited to record their responses to predefined employment interview questions on camera and submit them via an online platform. As a next step, one or more raters evaluate the single interview question video segments with regard to predefined criteria drawn from a job analysis. Previous research on “thin slices” has shown that people or automated cue detection can make valid judgments about states or traits of complete strangers based on short video segments of behavior samples (Ambady and Rosenthal, 1992; Borkenau et al., 2004; Carney et al., 2007; Batrinca et al., 2011). In the hiring and job-related domain, previous studies revealed that such thin slices are predictive for negotiation outcomes (Curhan and Pentland, 2007) and hirability impressions (Nguyen and Gatica-Perez, 2015).

With regard to research on applicant reactions, most studies have been based on the model proposed by Gilliland (1993), which evaluates applicants’ reactions from an organizational justice perspective. This model proposes that applicants’ overall perception of fairness depends on several personal and situational factors. This fruitful theoretical foundation has resulted in a large body of literature that has accumulated during the last two decades, including numerous single studies (Gilliland, 1994; Steiner and Gilliland, 1996; Bauer et al., 1998; Madigan and Macan, 2005), reviews (Ryan and Ployhart, 2000), meta-analyses (Hausknecht et al., 2004; Anderson et al., 2010), and theoretical refinements (Anderson, 2011). Even though a number of studies have investigated applicant reactions to new technology from the organizational justice perspective (Bauer et al., 2004, 2006), alternative approaches that can explain applicants’ higher or lower acceptance of new assessment technologies have rarely been addressed. Ryan and Ployhart (2000) criticized this narrow focus on the organizational justice perspective and suggested considering alternative paradigms such as the theory of reasoned action (Ajzen and Fishbein, 1977) or the theory of planned behavior (TPB; Ajzen, 1991). In the field of information system literature (Davis, 1989; Venkatesh and Davis, 2000; Venkatesh et al., 2003), theories that rely on the principles of the theory of reasoned action or planned behavior (Davis, 1989; Venkatesh and Davis, 2000; Venkatesh et al., 2003) have demonstrated their explanative value very well (King and He, 2006). Nevertheless, to the best of our knowledge, they have been neglected by research on applicant reactions to new technology, even if preliminary research has suggested its utility in predicting familiar topics, such as job seekers’ intentions to use job-search websites (Lin, 2010). Therefore, findings from research on technology acceptance may also allow for a better understanding of applicant reactions to new technology in selection.

The role of applicants’ interindividual differences and personality has been addressed as an additional source of variance for explaining reactions to traditional selection techniques in the past. This research seems to suggest that personality plays little role in explaining applicant reactions. For example, Hausknecht et al. (2004) described only zero to low meta-analytic correlations between conscientiousness, neuroticism, and perceptions of procedural justice. Nevertheless, Chan and Schmitt (2004) proposed that personal characteristics might be highly relevant to reactions to new technology (e.g., if applicants scoring high on neuroticism are more concerned with data security). Bauer et al. (2006) found that procedural justice perception moderates the relation between privacy concerns and test-taking motivation as well as organizational attractiveness. On these grounds, personal characteristics may provide further information in explaining applicants’ reactions to new technology.

The present study aimed to integrate findings from technology acceptance research with research on applicant reactions to new technology for the new selection procedure of asynchronous video interviewing. In addition, we addressed whether measures of personality could offer a better understanding of individual differences in applicants’ reactions to new technology. To our knowledge, this is the first study to integrate ideas from technology acceptance research and personality to predict applicants’ reactions to asynchronous video interviewing in personnel selection.

According to Ryan and Ployhart (2000, p. 566), research on applicant reactions addresses “attitudes, affect, or cognitions an individual might have about the hiring process.” Within the Gilliland (1993) model, applicant reactions play a role in determining applicants’ perceptions of procedural justice rules, test type, a company’s human resource policy, and the behavior of human resource executives during the process. Perceptions of fairness are also influenced by people’s prior selection outcomes (experience) and outcomes after hiring (e.g., performance) as well as their self-perceptions (e.g., self-efficacy). Gilliland specified 10 procedural justice rules (and two potential ones) assigned to three categories (formal characteristics, explanation, interpersonal treatment). They proposed that five out of these 10 were influenced by the specific procedure presented: the perception of job relatedness, the opportunity to perform, consistency of administration, perceived feedback, and the possibility of having a two-way interaction. An updated model by Hausknecht et al. (2004) suggested four categories that might influence applicants’ perceptions of selection properties consisting of personal characteristics, perceived procedural characteristics, job characteristics, and organizational context. Furthermore, applicant perceptions have been found to influence relevant outcome variables including selection procedure performance, self-perception, attitudes toward the hiring organization, and work attitudes and behavior. The strongest impact on important outcome variables has been reported for face validity and perceived job relatedness (Hausknecht et al., 2004).

Several methodological approaches have been developed to assess applicant reactions. For example, Steiner and Gilliland (1996) developed a widely used questionnaire that evaluates seven dimensions of applicant reactions. Bauer et al. (2001) developed a factor analytically confirmed measure for Gilliland’s procedural justice rules, the Selection Procedural Justice Scale (SPJS). Kersting (2008) proposed a questionnaire in three versions that address either personality inventories, cognitive tests, or assessment centers covering six to eight dimensions, such as perceived controllability, estimated measurement quality, and face validity, in order to predict overall acceptance.

In the present study, we operationalized attitudes to asyn chronous video interviewing as a multidimensional construct that includes four theoretical dimensions. These were selected on the basis of their estimated relevance in order to evaluate asynchronous video interviewing as a new technology in preselection: appropriateness in preselection, opportunity or chance to perform, consistency of administration or the absence of discrimination (fairness), and informativeness, reflecting the availability of all relevant information. Within the literature, the terms fairness reaction (Steiner and Gilliland, 1996) and attitudes to selection (Marcus, 2003) are used interchangeably in independent studies that applied the same measures. In order to capture the construct of reactions, we choose the term attitudes in the sense of the definition of attitudes as an evaluative judgment of a psychological object (Ajzen, 2000). The psychological object addressed in this study is the procedure of asynchronous video interviewing.

### Technology Acceptance Research

A large body of research that may contribute to our understanding of reactions toward asynchronous video interviewing has been performed in the field of technology acceptance. According to Davis (1989, p. 320), research on technology acceptance addresses “what causes people to use or to reject information technology.”. Despite all theoretical refinements (Venkatesh and Davis, 2000; Venkatesh et al., 2003; Venkatesh and Bala, 2008), the technology acceptance model suggests that the fundamental variables in determining the acceptance of new technology are perceived usefulness and perceived ease of use. According to Davis (1989, p. 320), perceived usefulness refers to a subjective advantageously system usage; perceived ease of use refers to “the degree to which a person believes that using a particular system would be free of effort.” The technology acceptance model in its original version (Davis, 1989) proposes that according to these antecedents, perceived usefulness and perceived ease of use are the underlying belief structures that result in a positive attitude toward a new technology. This positive attitude then results in behavioral intentions and actual future system usage. A large body of literature (Davis et al., 1989; Venkatesh, 1999; Venkatesh and Davis, 2000; King and He, 2006) has provided robust empirical support for the technology acceptance model and its core components. Typically, perceived usefulness and perceived ease of use explain about 40% of the variance in intentions to use and actual usage (Venkatesh and Davis, 2000). The extended model that refers to the TPB by Ajzen (1991) and includes external determinants (e.g., subjective norm) and moderators (e.g., voluntariness) typically explains up to 60% of the variance in intentions to use and actual system usage (Venkatesh and Davis, 2000).

In line with research from the information systems acceptance literature (Davis, 1989; Venkatesh and Davis, 2000), we propose that the higher the degree to which a selection procedure contributes to a persons’ perception of usefulness in either a practical sense (e.g., easier application, higher time-efficiency) or in a broader sense (e.g., by allowing candidates to present themselves more authentically), it will be associated with the level of positive reactions to new technology. Similarly, perceived ease of use is also supposed to serve as a significant predictor of applicant reactions to new technology.

### Individual Differences

An alternative approach to understand applicant reactions to new technology is derived from personality psychology. Surprisingly, only a small amount of research has been published on the role of personality with regard to applicant reactions to new technology in recruiting (Hausknecht et al., 2004). In the following, we refer to findings that involve computer self-efficacy, job interview self-efficacy, and the Big Five personality factors, because they relate to various aspects of selection and linked work-related outcomes.

Compeau and Higgins (1995, p. 192) defined computer self-efficacy as “a judgment of one’s capability to use computer.” The construct represents an extension of the Bandura (1982) concept of self-efficacy as the agent that functions between the belief in one’s personal abilities and behavior in the specific domain of computer usage. Computer self-efficacy is positively associated with actual computer usage (Compeau and Higgins, 1995) and perceived ease of use (Devaraj et al., 2008). Therefore, we hypothesized that computer self-efficacy would predict attitudes toward asynchronous video interviewing.

Whereas general self-efficacy refers to optimistic self-beliefs in coping with a variety of difficult demands in life (Schwarzer et al., 1997), job interview self-efficacy reflects one’s estimated ability to succeed in a job interview situation (Sieverding and Ortner, unpublished manuscript). Self-efficacy in specific domains has been shown to predict coping in a number of challenging situations (Luszczynska et al., 2005; Lippke et al., 2009). Next, we expected persons possessing high levels of job interview self-efficacy to prefer interactive selection methods to a higher degree than non-interactive selection procedures such as online tests or personality inventories. In the case of this particular procedure, the procedure is a non-interactive one, but the video should be able to catch capabilities that are similar to those captured in face-to-face interviews better than other non-interactive procedures such as cognitive tests.

Openness to experience refers to a person’s disposition to be imaginative and autonomous. People who score high on this trait are also described as having the tendency to be unconventional and non-conforming (Costa and McCrae, 1992). Barrick et al. (2001) found a positive relation between openness and training performance, whereas Devaraj et al. (2008) found no relation between openness and perceived usefulness. For openness, we postulated that people who possess higher levels of openness will be able to adapt more easily to the introduction of new technology. This is based on the assumption that this trait allows people to recognize the usefulness of a new technology, thus leading to a more positive attitudinal acceptance as proposed by the technology acceptance model. Furthermore, we expected that persons low on this trait might have more negative attitudes toward asynchronous video interviewing based on finding it difficult to feel comfortable with regard to their attitudes because people low on openness tend to be more conventional and conforming, even if they recognize the usefulness of the technology on a cognitive level.

People with higher levels of conscientiousness are described as dutiful, ambitious, diligent, and they take action to improve their job performance (Costa and McCrae, 1992). In the context of technology acceptance, Devaraj et al. (2008) argue that conscientiousness reflects an intrinsic motivation for further job achievement and is therefore related to perceived usefulness and intentions to system usage. In line with these postulates, in fact, Hausknecht et al. (2004) reported a small relation between conscientiousness and procedural justice perception. Conscientiousness has also been found to be associated with other job-related domain outcomes such as leadership (Judge et al., 2002a), job satisfaction (Judge et al., 2002b), and work performance (Barrick et al., 2001). Based on previous findings on effects of applicants’ conscientiousness, we propose that higher levels of this trait should be related to positive attitudes because people with high conscientiousness associate new technology with potential future job achievement.

Extraversion refers to a tendency to be sociable, vivacious, spirited, and to experience positive affect. Extraverted people are also described as being optimistic (Costa and McCrae, 1992). Extraversion has been shown to be associated with several job-related domains such as leadership (Judge et al., 2002a) or performance appraisal (Judge and Erez, 2007). In the context of technology acceptance, Devaraj et al. (2008) argued that persons possessing higher levels of extraversion show higher usage intentions because they expect a subsequent gain in positive social consequences from system usage compared to persons possessing lower levels of extraversion. Extraversion has also been shown to be associated with impression management and self-presentation in social networks (Krämer and Winter, 2008). Referring to previous findings on extraversion, we expect that a video-based selection tool will provide extraverted people with more opportunities for impression management than other processes at the same stage of the selection process (e.g., cognitive tests) compared to people low on this trait.

Neuroticism describes a tendency of people to adjust poorly to negative affect to perceive high levels of stress and to be anxious, and insecure (Costa and McCrae, 1992). Bauer et al. (2006) addressed the issue of privacy concerns in applicant reactions in the field of online hiring processes, but did not address neuroticism as a trait, which might likely to be associated with privacy concerns (Chan and Schmitt, 2004). In the work-related domain, Judge et al. (2002b) found that neuroticism was negatively related to job satisfaction. Truxillo et al. (2006) found a significant negative relation between neuroticism and the perception of social fairness after test taking. In line with findings regarding neuroticism, we argue that people who feel stress as a result of being challenged by an unfamiliar assessment situation and or who might be concerned about the safety of their personal data will have less positive attitudes toward a new video assessment tool.

The present research aims to study several predictors for attitudes toward asynchronous interviewing. In detail, we aim to clarify the following research questions regarding applicant reactions toward a particular new selection technology:

(a) Are perceived usefulness and perceived ease of use significant predictors for applicants’ attitudes toward asynchronous video and if these effects are incremental to individual differences?
(b) Do computer self-efficacy and job interview self-efficacy significantly predict attitudes toward asynchronous video interviewing?
(c) Will extraversion and conscientiousness be positively and neuroticism negatively associated with attitudes toward asynchronous video interviewing?
(d) Will openness to experience moderate the relation between perceived usefulness and attitudes toward asynchronous video interviewing?

## Materials and Methods

### Participants

One hundred six adults (65 women, 41 men) aged 18 to 51 (*M* = 22.46, *SD* = 4.33) voluntarily participated in this study and were recruited either from two medium-sized German universities (Free University Berlin, University of Potsdam). Psychology majors comprised 48.88% of the sample; others were students from very different subjects such as business, economics, or natural science. Participants received either credit toward an introductory psychology class or 10€ (approximately $11 US).

### Procedure

Participants completed the study online. First, questionnaires (computer self-efficacy, job interview self-efficacy, Big Five) were presented. Next, participants were required to read the following text before they were directed to the video interviewing platform:

Please imagine the following situation: You have applied for an attractive job in another city (e.g., Munich, Hamburg). You receive an invitation via email to participate in an online asynchronous video interview. A link is attached to this email. Please note that this procedure is part of a preselection process because many people have applied for the same job across the country. The goal of the procedure is to obtain a personal impression of all applicants, in addition to their resumes, to help the hiring company make a well-informed decision about which of the applicants to invite for a personal employment interview at the company’s headquarters. This procedure is not intended to substitute for a personal (face-to-face) interview.

Participants were directed to the video platform where they were required to complete a mock asynchronous video interview that included three questions extracted from typical employment interviews. Afterward, participants completed a questionnaire including scales about their judgments of their performance, how seriously they took the interview, perceived usefulness, perceived ease of use, favorability rating, and procedural justice dimensions. Demographic questions were completed last.

### Materials

#### Computer Self-Efficacy

Computer self-efficacy was measured with a six-item scale adapted from a German translation of the computer user self-efficacy scale (Cassidy and Eachus, 2002; Spannagel and Bescherer, 2009). Responses to these items were scored on a Likert scale ranging from 1 (disagree strongly) to 5 (agree strongly). Three of the six items were reverse coded. To create the scale, the reverse-coded items were recoded and the six items were averaged. Cronbach’s alpha was α = 0.91.

#### Job Interview Self-Efficacy

Job interview self-efficacy was measured using a five-item scale (α = 0.82). The items were selected from (Sieverding and Ortner, unpublished manuscript). The items were scaled from 1 (disagree strongly) to 5 (agree strongly). One of these items was reverse coded.

#### Big Five

The Big Five personality dimensions were assessed using self-ratings based on unipolar adjectives (four adjectives per dimension). Each adjective was rated on a 5-point scale ranging from 1 (not at all) to 5 (a lot). These were taken from a German list of trait descriptors, which were collected and factor analyzed by Ostendorf (1990). Internal consistencies were sufficient with α = 0.72 for extraversion, α = 0.74 for agreeableness, α = 0.69 for neuroticism, α = 0.75 for openness, and α = 0.85 for conscientiousness.

#### Perceived Usefulness

Perceived usefulness was measured by six items inspired from the scale by Davis (1989), which were adapted for the asynchronous interviewing tool. Each item was rated from 1 (disagree strongly) to 5 (agree strongly). The reliability of this scale was α = 0.75.

#### Perceived Ease of Use

Perceived ease of use was assessed by a six-item Likert-type scale that was adapted for the tool used in this study and translated from the scale by Davis (1989). The items were rated on a scale ranging from 1 (totally disagree) to 5 (totally agree). One item was reverse coded. The internal consistency was α = 0.88.

#### Attitudes Toward Asynchronous Video Interviewing

Attitudes toward asynchronous video interviewing were assessed with a 12-item Likert-type scale ranging from 1 (disagree strongly) to 5 (strongly agree) based on acceptance research and the literature on attitudes toward selection procedures (Steiner and Gilliland, 1996; Bauer et al., 2004; Kersting, 2008). Three items each covered four content domains appropriateness, chance to perform, fairness, and informativeness. The internal consistency was acceptable at α = 0.81. A translation of the Steiner and Gilliland (1996) questionnaire was used to provide additional descriptive results for the literature. The correlations between our attitudes measure and the Steiner and Gilliland questionnaire revealed *r* = 0.65, *p* < 0.001 for the two-item favorability construct (α = 0.67) and *r* = 0.74, *p* < 0.001 for all averaged ratings on the seven procedural justice dimensions (α = 0.76).

#### Control Variables

In line with previous research and the well-understood relation between selection-process outcomes (e.g., being hired) and a positive evaluation of the selection method (Bauer et al., 1998; Ryan and Ployhart, 2000; Chan and Schmitt, 2004; Hausknecht et al., 2004), we assume to find this relationship also in the case of asynchronous video interviewing. If immediate feedback is not available, which is the case in asynchronous video interviewing, self-rated performance has been revealed to serve as a basis for evaluation (Hausknecht et al., 2004). Thus, we included self-rated performance in our analyses. Self-rated performance was assessed by two items asking “How would you rate your performance in the video interview with regard to your rhetoric and verbal competencies?” and “…with regard to your conceptual and analytic competencies?” The items were rated on a 5-point Likert scale ranging from 1 (not good at all) to 5 (very good). The two items correlated with *r* = 0.61, *p* < 0.001 and were averaged. As further control variables, we collected information on gender, age, mother tongue, prior experience with selection methods, and how seriously the participants took the video interview to have the chance to exclude individual participants from further analyses. Seriousness was assessed by the item “How seriously did you take the interview compared to if you had really applied for a job?” and answered on a 5-point scale ranging from 1 (not seriously at all) to 5 (as seriously as in a real interview). We did not include an additional questionnaire about the familiarity of participants with technology, because the computer self-efficacy construct already reflects variations of previous mastery experience in the technology domain (Compeau and Higgins, 1995). Moreover, no participant reported previous experience with asynchronous video interview technology.

#### Interview and Survey Application

The online platform for the asynchronous video interview was the interview suite of the German provider viasto^1^. For questionnaires, we used the web-based software of SoSci Survey^2^.

## Results

**Table 1** presents a summary of means, standards deviations, intercorrelations, and internal consistencies for all study variables. The data were analyzed using R version 3.1.2 for Macintosh (R Core Team, 2014). To test our research questions, a moderated hierarchical regression analyses was computed to predict attitudes toward asynchronous video interviewing. As recommended by Aiken and West (1991), all continuous variables were centered. As depicted in **Table 2**, we entered first age, gender, and self-rated performance, which served as control variables. In step 2, computer self-efficacy, job interview self-efficacy, and the four relevant Big Five personality variables were entered. Next, in step 3, the technology acceptance model variables perceived usefulness and perceived ease of use were entered. The interaction term of perceived usefulness and openness to experiences was entered last. The results are presented in detail from the block that explained most of the variance and significantly more than the others.

**Table 1:** Means, standard deviations, and intercorrelations for study variables.

		*M*	*SD*	1	2	3	4	5	6	7	8	9	10	11	12	13
(1)	Age	22.46	4.03	-												
(2)	Gender (1 = female)	1.39	0.49	0.11	-											
(3)	Self-rated performance	2.86	0.74	-0.06	0.12	*0.76*										
(4)	Computer self-efficacy	3.96	0.77	0.12	0.36^∗∗∗^	0.16	*0.91*									
(5)	Job interview self-efficacy	3.57	0.58	0.03	0.09	0.14	0.26^∗∗^	*0.82*								
(6)	Openness	3.80	0.51	-0.01	0.22^∗^	0.00	0.21^∗^	0.31^∗∗^	*0.75*							
(7)	Conscientiousness	3.92	0.74	0.03	-0.18	0.09	0.00	0.18	0.17	*0.85*						
(8)	Extraversion	3.87	0.66	-0.20^∗^	-0.24^∗^	-0.07	-0.15	0.20^∗^	0.12	0.02	*0.72*					
(9)	Agreeableness	4.08	0.55	0.07	-0.05	-0.01	0.07	0.05	0.05	0.01	0.13	*0.74*				
(10)	Neuroticism	3.29	0.79	-0.06	-0.47^∗∗∗^	-0.16	-0.45^∗∗∗^	-0.21^∗^	-0.06	0.16	0.04	-0.10	*0.69*			
(11)	Perceived usefulness	3.39	0.65	0.03	-0.02	0.08	0.15	-0.05	0.02	0.11	0.00	-0.04	-0.01	*0.75*		
(12)	Perceived ease of use	4.19	0.72	0.09	0.06	0.19	0.30^∗∗^	0.18	0.13	0.08	0.01	0.02	-0.17	0.09	*0.88*	
(13)	Attitudes toward asynchronous video interviewing	3.18	0.70	-0.05	0.03	0.02	0.25^∗^	0.18	0.09	0.25^∗∗^	0.13	-0.10	-0.12	0.68^∗∗∗^	0.25^∗^	*0.81*


**N* = 96–106 due to missing data. Cronbach’s alpha values are depicted in italics on the diagonal. ^∗^*p* < 0.05, ^∗∗^*p* < 0.01, ^∗∗∗^*p* < 0.001.*

**Table 2:** Hierarchical regression analysis predicting attitudes toward asynchronous video interviewing.

	Step 1		Step 2		Step 3		Step 4	
				
	β	*t*	β	*t*	β	*t*	β	*t*
Age	-0.08	-0,86	-0.08	-0.88	-0.12	-1.62	-0.12	-1.77
Gender	0.01	0.13	-0.01	-0.11	0.06	0.65	0.06	0.73
Self-rated performance	0.26	2.74^∗∗^	0.25	2.64^∗∗^	0.08	0.99	0.08	1.04
Computer self-efficacy			0.25	2.30^∗^	0.10	1.21	0.08	1.00
Job interview self-efficacy			-0.01	-0.12	0.11	1.37	0.12	1.49
Openness			0.00	-0.04	0.01	0.14	0.03	0.36
Conscientiousness			0.23	2.34^∗^	0.13	1.75	0.14	1.87
Extraversion			0.19	1.91	0.15	1.97	0.13	1.65
Neuroticism			-0.05	-0.45	-0.05	-0.58	-0.04	-0.42
Perceived usefulness					0.62	8.45^∗∗∗^	0.62	8.67^∗∗∗^
Perceived ease of use					0.13	1.61	0.15	2.01^∗^
Perceived usefulness^∗^Openness							0.22	2.61^∗^
*R*^2^		0.07		0.21^∗∗^		0.56^∗∗∗^		0.59^∗∗∗^
Δ*R*^2^ step				0.14^∗^		0.34^∗∗∗^		0.03^∗^
*F*		2.62		2.90^∗∗^		10.73^∗∗∗^		11.01^∗∗∗^


**N* = 106. ^∗^*p* < 0.05, ^∗∗^*p* < 0.01, ^∗∗∗^*p* < 0.001.*

**FIGURE 1:**
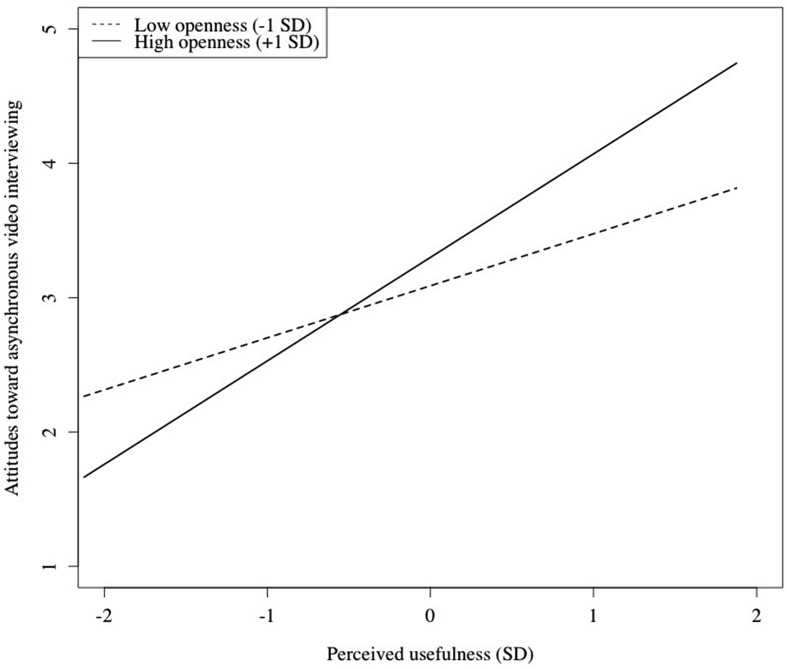
**Moderating effect of openness on the relationship between perceived usefulness and attitudes toward asynchronous video interviewing**.

As shown in **Table 2**, the hierarchical regression analysis regarding attitudes toward asynchronous video interviewing yielded highly significant results [*R*^2^ = 0.59, *F*(12,93) = 11.01, *p* < 0.001]. Control variables accounted for 7% (*ns*) of variation, individual differences for another 14% (*p* < 0.01), perceive usefulness and perceived ease of use for additional 34% (*p* < 0.001), and the interaction between perceived usefulness and openness accounted for another 3% (*p* < 0.05) of variation in attitudes toward asynchronous interviewing. Thus, detailed results of the significant effects are reported for the fourth block: Perceived usefulness (β = 0.62. *p* < 0.001), and perceived eased of use (β = 0.15, *p* < 0.05) predicted significantly attitudes toward asynchronous video interviewing. A positive interaction emerged between perceived usefulness and openness (β = 0.22, *p* < 0.05). The simple slope analysis of this interaction term is presented in **Figure 1**. In the last block, no effects were revealed for gender, age, self-rated performance, computer self-efficacy, job interview self-efficacy, openness, conscientiousness, extraversion, and neuroticism.

## Discussion

In the present study, we aimed to increase knowledge about a newly developed technology for personnel selection. In order to highlight different aspects of applicants’ attitudinal reactions, which are strongly associated with technological acceptance in the terms of the TPB (Ajzen, 1991), we included technology acceptance predictors as well as personality aspects to explain variation in attitudes toward asynchronous video interviewing. First, and in line with our expectations, our findings support the applicability of the technology acceptance approach with regard to the new selection technology we investigated. In line with previous research (King and He, 2006; Lin, 2010), our data revealed that perceived usefulness and perceived ease of use were significant predictors of attitudes toward this new technology in personnel selection. This result confirms that recruiting technology must be easy to use and that at least some aspect of it must be perceived as useful by the applicant; for example, theoretically allowing for applications to be submitted 24 h a day and from around the world or offering potential employers a more authentic impression of an applicant’s abilities compared to other screening tools.

Our analyses regarding the relation between personality and attitudes toward this new recruiting technology revealed the expected positive relation between conscientiousness and attitudes toward the selection procedure only in parts. In line with previous research data (Hausknecht et al., 2004; Truxillo et al., 2006), higher conscientiousness in applicants was related to a more positive attitude toward this selection technique, but not anymore when we entered perceived usefulness and perceived ease of use. As proposed by Chan and Schmitt (2004), our results highlight the potential value of taking a look at the role of individual differences in the field of new technology even if there are no effects in the literature reported, yet. Furthermore, this study revealed that openness had a moderating effect on the relation between perceived usefulness and attitudes toward asynchronous video interviewing. This finding emphasizes the importance of individual differences in applicants’ abilities to adapt to new technology in personnel selection and may encourage future research to stop looking exclusively at linear relations between this trait and specific outcome variables. Nevertheless, we were not able to confirm a relevant relation for computer self-efficacy, job interview self-efficacy, extraversion, and neuroticism.

Job interview self-efficacy failed to significantly predict reactions toward this technology-based selection procedure. Effects of the simulated context and a lack of consequences from the given interview may serve as possible explanations for this result. Participants may not have been thinking of being evaluated as would usually occur after an interview, leading to a more optimistic self-view of their own capacities. Furthermore, the anonymous situation may also have facilitated an optimistic self-view regarding self-efficacy. As shown in studies regarding self-estimated intelligence, for example, people tend to provide a more accurate picture of themselves when they know they will receive feedback (Holling and Preckel, 2005). Hence, the specific questions from the job interview self-efficacy questionnaire may have been more related to face-to-face job interview situations, depending on interactive elements not given in the asynchronous technology. Computer self-efficacy also did not have revealed a significant effect in predicting attitudes toward asynchronous video interviewing. People who possessed stronger beliefs in their ability to use a computer did not hold more positive attitudes toward this technology-enhanced selection tool. Future studies may reveal whether this lack of effect is generalizable to other subpopulation. Regarding neuroticism, again, the lack of an actually stressful situation may have diminished concerns about data security and may have diminished the potential for a maladaptive tendency to emerge in persons possessing higher levels of neuroticism. Nevertheless, the use of simulated studies based on student samples is common in the investigation of applicant reactions (Bauer et al., 2004).

Regarding age and gender, the data failed to significantly predict attitudes toward asynchronous video interviewing. This results was contrary to previous finding from the technology acceptance model research that revealed both gender and age differences in technology adaption (Venkatesh and Morris, 2000; Renaud and Van Biljon, 2008), but it was in line with findings from the selection domain, where Hausknecht et al. (2004) report meta-analytic correlation at or near zero for personal characteristics such as age and gender with applicant perceptions. For self-rated performance, again, the data failed to significantly predict attitudes toward this specific selection procedure. One possible explanation for this result may lie in the fact that the participants in our study lacked an applicable frame of reference. Chan and Schmitt (2004) noted that a frame of reference shaped by past experience may be relevant for such judgments (e.g., when students are familiar with standardized tests from school). It will, however, be important to replicate these results in the future with a less relatively homogeneous sample and in a real-life setting.

Last, we provided preliminary data to add to the cumulative body of literature on the classification of popular selection procedures by their favorability among applicants with regard to the selection tool of asynchronous video interviewing. Participants’ ratings led to a mean score for the favorability of asynchronous video interviewing technology (*M* = 4.00, *SD* = 1.28) that was in the range that previous research classified as a favorably evaluated selection procedure (Anderson et al., 2010). For future meta-analysis, we will provide all means, standard deviations, and intercorrelations of single items of the questionnaire by Steiner and Gilliland (1996) as Appendix (see **Table A1**).

It is important for practitioners to understand the factors that affect applicant reactions to new technology in personnel selection. To summarize our results and connect them to basic research approaches in this field, our results added two more variables to the well-examined organizational justice framework, and these two variables are import for reactions to innovative selection tools. Like every other professional information systems, new technology in recruiting has to be easy to use and has to be useful in a way that goes beyond existing applications (Venkatesh and Davis, 2000; King and He, 2006; Lin, 2010). Our results might also encourage researchers to test alternative theoretical frameworks to explain applicant reactions to new technology, i.e., theories derived from a media richness perspective (Potosky, 2008). With regard to asynchronous video interviewing technology as a selection tool, our preliminary data enabled us to classify the favorability of this specific selection procedure in comparison to alternative selection techniques at the same stage of the selection process. We found that asynchronous video interviewing was in the same range as other non-interactive procedures like personality inventories or cognitive tests, which are applied as online tools at the same stage of the selection process (Marcus, 2003; Anderson et al., 2010).

Several limitations of the current study require further research on the topic. First, this study assessed applicant reactions in a hypothetical context. The meta-analysis by Hausknecht et al. (2004) found significant differences in the strengths of effect sizes between simulated and real-life studies, but the study did not find differences in the directionality. Nevertheless, future research should strive to replicate these findings in a real-life context. Second, the study focused on the link between perceived usefulness, perceived ease of use, and reactions toward asynchronous video interviewing, but did not capture one important other component of the TPB: subjective norm. Anderson et al. (2010) reported that applicants’ perception of a tool’s distributive rate is highly related to the favorability of a selection procedure. The technology addressed by this study is not wide spread yet, but longitudinal studies could examine whether the distribution rate (people’s beliefs that it is a standard procedure) affects favorability or any other reaction. With respect to the behavioral control belief structure of the TPB, we assessed computer self-efficacy as well as job interview self-efficacy. Third, the sample was drawn from a relatively homogeneous sample with regard to demographic variables and education (students). This could also cause restriction of variance with regard to other characteristics such as computer experience or personality traits. Last, the sample was cross-sectional. A variety of scholars of applicant reactions have stated (Chan and Schmitt, 2004) that there is a need to consider applicant reactions over time. Thus, more research is required here.

The study presented here offers new insights into the determinants of applicants’ reactions to new technology in personnel selection and demonstrated that perceived usefulness and perceived ease of use are important predictors for reactions toward technology-based selection procedures. These findings also offer new insights into the role of personality for reactions to new technology in recruiting, especially with regard to openness. Last, the present data offered initial descriptive results about the favorability of the new technology of asynchronous video interviewing compared to other methods.

## Author Contributions

All three authors developed the study concept. FB programmed the web based questionnaires and carried out the data collection and performed the data analysis. FB drafted an initial version of the manuscript that was refined and revisited successively by TO and DF until this final draft.

## Conflict of Interest Statement

The authors declare that the research was conducted in the absence of any commercial or financial relationships that could be construed as a potential conflict of interest.

## Footnotes

1 www.viasto.com

2 www.soscisurvey.de

## APPENDIX

**Table A1:** Means, standard deviations, and intercorrelations (Pearson) of the Steiner and Gilliland (1996) Questionnaire.

	Variable	*M*	*SD*	1	2	3	4	5	6	7	8
(1)	Overall favorability	4.00	1.28	0.67							
(2)	Scientific Evidence	3.48	1.27	0.32^∗∗^	-						
(3)	Face validity	4.37	1.15	0.48^∗∗^	0.49^∗∗^	-					
(4)	Chance to perform	3.53	1.41	0.40^∗∗^	0.41^∗∗^	0.43^∗∗^	-				
(5)	Interpersonal warmth	3.59	1.88	0.40^∗∗^	0.27^∗∗^	0.26^∗∗^	0.15	-			
(6)	Employer’s rights	4.35	1.65	0.39^∗∗^	0.38^∗∗^	0.45^∗∗^	0.35^∗∗^	0.12	-		
(7)	Respectful of privacy	4.77	1.67	0.42^∗∗^	0.43^∗∗^	0.49^∗∗^	0.38^∗∗^	0.22^∗^	0.53^∗∗^	-	
(8)	Widely used	2.76	1.42	0.31^∗∗^	0.24^∗^	0.33^∗∗^	0.23^∗^	0,18	0.37^∗∗^	0.23^∗^	-

**N* = 97–106 due to missing data. ^∗^*p* < 0.05, ^∗∗^*p* < 0.01.*
